# Earth as a Topical Application in Surgery

**Published:** 1872-06

**Authors:** 


*Earth as a Topical application in Surgery.* By ADDINELL HEWSON, M. D. With four photo-relief illustrations.

In 1869 Dr. Hewson, whose attention had been previously attracted to the disinfecting properties of earth as illustrated by the earth closet, conceived the idea of making use of this substance as a topical application to all cases needing such application which should come under his care during his term of service in the surgical ward of the Pennsylvania Hospital.

After making a thorough trial for six months of this dressing, in cases many of them which were as severe as usually fall under the observation of medical men Dr. Hewson was satisfied as to the value and utility of earth as a topical dressing in surgical cases.

Fearing however that he might be accused of being too hasty in his conclusions, he has allowed nearly three years to give his observations the benefit of further research and study. The result of this has been the production of a work showing the results of the application of earth in ninety-three cases, together with comments on the effects of the contact of earth, and modus operandi of earth, as a deoderizer and over puterfaction, in its effect on Living Parts.

We have been much interested in reading Dr. Hewson's book and shall look with interest for any further light, which may be thrown on the subject. The four illustrations which the book contains are finely executed and show a great advance in the art of illustrating books which we hope to see more fully introduced.

